# High‐Fat Diet–Induced Metabolic Stress Durably Reprograms the Liver Regenerative Compartment Toward a Senescence‐Like State

**DOI:** 10.1111/acel.70769

**Published:** 2026-10-16

**Authors:** Mirai Yamazaki, Hiroya Yamada, Eiji Munetsuna, Yoshitaka Ando, Genki Mizuno, Takuya Wakasugi, Tomohide Sakakibara, Kumpei Hada, Hayato Ichikawa, Ryoga Senga, Moeno Asai, Hiroaki Ishikawa, Koji Suzuki, Koji Ohashi, Nobutaka Ohgami

**Affiliations:** ^1^ Department of Hygiene Fujita Health University School of Medicine Toyoake Japan; ^2^ Department of Animal Science and Biotechnology Azabu University School of Veterinary Medicine Sagamihara Japan; ^3^ Department of Medical Technology Yokkaichi Nursing and Medical Care University School of Nursing and Medical Care Yokkaichi Japan; ^4^ Department of Medical Technology Tokyo University of Technology School of Health Sciences Ota Japan; ^5^ Department of Informative Clinical Medicine Fujita Health University School of Medical Sciences Toyoake Japan; ^6^ Department of Preventive Medical Sciences Fujita Health University School of Medical Sciences Toyoake Japan

**Keywords:** cellular senescence, liver progenitor, liver regeneration, metabolic dysfunction‐associated steatotic liver disease (MASLD), metabolic stress, mitochondrial dysfunction, organoid, stem cell aging

## Abstract

Chronic metabolic stress is increasingly recognized as a driver of aging‐like tissue dysfunction, but whether it durably reprograms liver regenerative cells, potentially compromising tissue maintenance and repair, remains unknown. Here, we show that high‐fat diet (HFD)–induced metabolic stress promotes a persistent senescence‐like state in the liver regenerative compartment. In male rats, chronic HFD feeding induced hepatic steatosis together with oxidative stress, inflammatory responses, and senescence‐associated changes. Following carbon tetrachloride‐induced liver injury, HFD‐fed animals exhibited reduced proliferative responses. To determine whether these abnormalities were durably retained within regenerative cell populations, we established liver organoids from control and HFD‐fed rats. HF‐derived organoids showed reduced formation efficiency and impaired serial propagation capacity outside the original metabolic environment. Senescence‐associated abnormalities, including increased senescence‐associated β‐galactosidase staining, reduced Lamin B1 staining, and decreased Ki67 positivity, remained detectable through Passage 3. Transcriptomic and functional analyses revealed mitochondrial dysfunction and enhanced oxidative and inflammatory responses. These organoids also exhibited enhanced lipid accumulation and inflammatory secretion following fatty acid challenge. Treatment with the mitochondria‐targeted antioxidant MitoQ reduced oxidative stress and inflammatory secretion, improved oxygen consumption, and partially restored organoid formation efficiency, supporting a functional contribution of mitochondrial dysfunction to regenerative impairment. Together, these findings support a model in which chronic metabolic stress leaves persistent senescence‐like dysfunction within the liver regenerative compartment, extending its consequences beyond abnormalities in mature liver tissue.

## Graphical abstract

High‐fat diet‐induced metabolic stress durably reprograms regenerative liver cells into a senescence‐like phenotype. The state is characterized by lipid accumulation, mitochondrial dysfunction, oxidative stress, inflammation, and impaired regenerative capacity. Aging‐like hepatic dysfunction under metabolic stress is therefore the result of persistent aging programs embedded within the regenerative cell population.

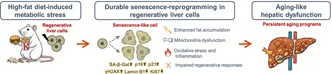

## 1 Introduction

Chronic metabolic stress is increasingly recognized as a driver of aging‐like tissue dysfunction across multiple organs (López‐Otín et al. 2023). In the liver, persistent nutrient excess and obesity‐associated metabolic disturbance promote not only steatosis, but also mitochondrial dysfunction, oxidative stress, chronic low‐grade inflammation, and impaired regenerative capacity (DeAngelis et al. 2005; Ogrodnik et al. 2017; Satapati et al. 2015). These features closely resemble core aspects of hepatic aging, suggesting that prolonged metabolic stress can induce an aging‐like state in the liver independent of chronological age (Horvath et al. 2014). Thus, chronic metabolic stress may compromise tissue maintenance and repair beyond its immediate effects on lipid accumulation.

Cellular senescence is a central hallmark of aging and a major contributor to chronic tissue dysfunction. Senescent cells are characterized by persistent cell‐cycle arrest and acquisition of a pro‐inflammatory secretory phenotype that disrupts tissue homeostasis (Campisi and D'Adda Di Fagagna 2007). In metabolically stressed liver, senescence‐associated changes are well established and have been linked to mitochondrial dysfunction, oxidative stress, inflammatory signaling, and impaired regeneration (Dabravolski et al. 2021; Ogrodnik et al. 2017; Timchenko 2009). However, these studies have focused predominantly on mature parenchymal cells, particularly hepatocytes. Tissue‐resident stem and progenitor cells are essential for long‐term tissue maintenance and repair, and their functional decline is a fundamental feature of aging (Oh et al. 2014). Across multiple organs, aging‐associated dysfunction of these regenerative populations compromises self‐renewal and regenerative competence, can alter the functional properties of their progeny, and may promote a pro‐inflammatory tissue environment, thereby propagating tissue dysfunction (Ambrosi et al. 2021; Funk et al. 2023; Oh et al. 2014; Stearns‐Reider et al. 2017). Metabolic stressors such as obesity and chronic overnutrition also impair stem/progenitor function in several tissues (Alessio et al. 2020; Haller and Jasper 2016; Wu et al. 2013), raising the possibility that chronic metabolic stress similarly affects the liver regenerative compartment. However, whether chronic metabolic stress durably reprograms this compartment, establishing a persistent senescence‐like state that is maintained beyond the original metabolic environment, remains unknown.

Organoids are self‐organizing three‐dimensional cultures derived from tissue‐resident stem or progenitor cells that recapitulate key features of tissue regeneration and disease (Zhao et al. 2022). Organoids derived from aged tissues show reduced formation efficiency, impaired regenerative potential, senescence‐associated features, and age‐related molecular alterations that can be retained ex vivo (Funk et al. 2023; Hu et al. 2018; Lewis et al. 2020; Uchida et al. 2018). Because organoids can be propagated outside the original tissue environment, they provide a means to distinguish transient responses maintained by the metabolic milieu from persistent alterations retained within organoid‐forming regenerative cell populations. Accordingly, liver organoids provide a useful system for determining whether metabolic stress induces durable reprogramming of the liver regenerative compartment that persists independently of the original metabolic environment.

In the liver, facultative regenerative programs can contribute to epithelial repair under defined injury conditions (Köhn‐Gaone et al. 2016), highlighting the potential importance of maintaining regenerative competence. We therefore asked whether chronic high‐fat diet (HFD)–induced metabolic stress promotes a durable senescence‐like state within this compartment, potentially contributing to persistent hepatic dysfunction. To address this question, we assessed senescence‐associated alterations in the liver and regenerative responses after CCl_4_‐induced injury and established liver organoids from control and HFD‐fed rats to determine whether these abnormalities persisted ex vivo during serial propagation. We further used pharmacological intervention to examine the functional contribution of mitochondrial dysfunction to impaired organoid formation.

## 2 Materials and Methods

### 2.1 Animals

All procedures were approved by the Animal Experiment Committee of Fujita Health University and were conducted in accordance with the institutional guidelines (approval number: APU19096). Three‐week‐old male Sprague–Dawley rats (Shizuoka Laboratory Center, Shizuoka, Japan) were acclimatized for 1 week and housed two per cage under controlled conditions (23°C ± 3°C; 12‐h light/dark cycle). The rats were randomly assigned to either a control group (C group) fed a standard MF diet (Oriental Yeast, Tokyo, Japan) or a HFD (45% kcal from fat; Research Diets, New Brunswick, NJ, USA; HF group), and maintained with ad libitum access to food and water. Body weight and food intake were measured weekly. After 8 weeks, the animals were fasted for 6 h prior to sample collection. The rats were anesthetized by inhaling isoflurane, and samples were collected. Serum and liver samples were stored at −80°C until further analysis. Liver tissues used for organoid generation were processed immediately after dissection without freezing.

### 2.2 Serum Biochemical Analyses

Serum biochemical analyses were conducted as previously described (Munetsuna et al. 2021). Serum triglyceride (TG), total cholesterol (T‐Cho), and high‐density lipoprotein cholesterol (HDL‐C) levels were measured using enzymatic assay kits (Criogent; Sekisui Medical, Tokyo, Japan) with an automated biochemical analyzer (BiOLiS 24i; Tokyo Boeki Kikai, Tokyo, Japan), according to the manufacturer's instructions. Non–HDL cholesterol (non‐HDL‐C) levels were calculated by subtracting HDL‐C from T‐Cho.

### 2.3 Hepatic Lipid Measurements

Hepatic TG content was determined using the Folch method (Yamazaki et al. 2016). Briefly, liver tissues were homogenized in a 50 mM Tris–HCl, and total lipids were extracted. Lipid extracts were resuspended in a 10% Triton X‐100/isopropanol and were quantified using the Criogent TG kit with ARVO X5 (PerkinElmer, Waltham, MA, USA). TG levels were normalized to liver tissue weight (mg/g).

Hepatic lipid peroxidation (LPO) levels were measured by the thiobarbituric acid reactive substances (TBARS) assay. Liver tissues were homogenized in RIPA buffer, and supernatants were analyzed according to standard procedures. Fluorescence intensity was measured at 530/590 nm using ARVO X5. LPO levels were normalized to tissue weight (nmol/g).

### 2.4 Hematoxylin–Eosin and Oil Red O Staining

Liver tissues were fixed in 4% paraformaldehyde for 24 h, cryoprotected with 30% sucrose, and embedded in O.C.T. Compound (Sakura Finetek Japan, Tokyo, Japan). Sections were cut using a cryostat (CM1950; Leica Microsystems, Wetzlar, Germany) at 3–5 μm for hematoxylin and eosin (HE) staining and 10 μm for Oil Red O staining.

For HE staining, the sections were stained with hematoxylin for 5 min and eosin Y for 3 min. For Oil Red O staining, the sections were pretreated with 60% isopropanol. The sections were stained with Oil Red O for 15 min and mounted with an aqueous mounting medium (SouthernBiotech, Birmingham, AL, USA). Images were acquired using a BX43 (Olympus, Tokyo, Japan).

### 2.5 SA‐β‐Gal Staining and Quantification

SA‐β‐gal activity was assessed in liver and organoid cryosections as previously described (Jannone et al. 2020). Cryosections (15 μm thick) were fixed with 0.2% glutaraldehyde for 10 min and incubated in staining solution containing 40 mM citric acid/sodium phosphate buffer (pH 5.8), 5 mM potassium ferrocyanide, 5 mM potassium ferricyanide, 150 mM NaCl, 2 mM MgCl_2_, and 1 mg/mL X‐gal for 20 h at 37°C. Images were acquired using a BZ‐X800 microscope. SA‐β‐gal positivity was expressed as the percentage of positive area relative to the total analyzed area.

### 2.6 Immunohistochemical Analysis of Liver Sections

Liver tissues were fixed in 10% neutral‐buffered formalin for 48 h, embedded in paraffin, and sectioned at 4 μm. Following deparaffinization and antigen retrieval in 10 mM Tris–EDTA, sections were blocked with 1.5% normal goat serum. For immunohistochemistry, sections were incubated with mouse anti‐p16 (1:400; Santa Cruz Biotechnology, Dallas, TX, USA), rabbit anti‐p21 (1:2000; Proteintech, Rosemont, IL, USA), mouse anti‐γH2AX (1:2000; Santa Cruz Biotechnology), or rabbit anti‐Ki67 (1:800; Abcam, Cambridge, UK) followed by Histofine Simple Stain MAX‐PO (MULTI) and DAB visualization. For Lamin B1 immunofluorescence, sections were incubated with mouse anti‐Lamin B1 (1:100; Santa Cruz Biotechnology), followed by fluorescent secondary antibodies (Thermo Fisher Scientific, Waltham, MA, USA). Images were acquired using a BZ‐X800 microscope (Keyence, Osaka, Japan). Five random fields were analyzed per section. p16‐, p21‐, γH2AX‐, and Ki67‐positive cells were expressed as percentages of total nuclei, and Lamin B1 fluorescence intensity was normalized to the number of nuclei.

### 2.7 CCl_4_ Intoxication

Carbon tetrachloride (CCl_4_; Sigma‐Aldrich, Louis, MO, USA) was administered intraperitoneally at a dose of 1 mL/kg body weight twice within 1 week. CCl_4_ was diluted 1:1 (v/v) in olive oil immediately before injection. Two weeks after the final CCl_4_ injection, animals were sacrificed and their liver tissues were collected for immunohistochemical analysis.

### 2.8 Measurement of Inflammatory Cytokines

The concentrations of the inflammatory cytokines, including interleukin‐1β (IL‐1β), interleukin‐6 (IL‐6), tumor necrosis factor‐α (TNF‐α), C‐X‐C motif chemokine ligand 1 (CXCL1), and monocyte chemoattractant protein 1 (MCP‐1) were measured using a MILLIPLEX MAP Rat Cytokine/Chemokine Magnetic Bead Panel (Merck Millipore, Billerica, MA, USA) according to the manufacturer's instructions. Samples were analyzed using a Luminex‐based detection system (Luminex 100; Luminex Corporation, Austin, TX, USA). Hepatic cytokine levels were normalized to applied protein content. Cytokine concentrations in organoid culture supernatants were normalized to the total DNA content of the corresponding organoids to account for differences in organoid number.

### 2.9 Generation and Passaging of Rat Liver Organoids

Rat liver organoids were established as previously described in our study (Yamazaki et al. 2024). Briefly, freshly isolated liver tissues were cut into small fragments and digested with collagenase and dispase for 2 h. Dissociated cells were filtered through a 70‐μm cell strainer (Corning, NY, USA) and a 37‐μm reversible cell strainer (Stemcell Technologies, Vancouver, BC, Canada). Cells retained on the 37‐μm strainer were embedded in Matrigel (Corning) and plated as 50‐μL domes in 24‐well plates.

The organoids were cultured in Advanced DMEM/F12 (Thermo Fisher Scientific) supplemented with 50% WNT3a‐conditioned medium, 10% RSPO1‐conditioned medium, 100 ng/mL Noggin (PeproTech, Rocky Hill, NJ, USA), 50 ng/mL EGF (Thermo Fisher Scientific), 50 ng/mL HGF (PeproTech), and 10 nM gastrin (Sigma‐Aldrich), 100 ng/mL FGF10 (PeproTech), 10 mM nicotinamide (Wako Pure Chemicals, Osaka, Japan), 1.25 mM N‐acetylcysteine (Sigma‐Aldrich), 10 μM Y‐27632 (Wako Pure Chemicals), 25 mM HEPES (Thermo Fisher Scientific), B27 supplement without retinoic acid (Thermo Fisher Scientific), and N2 supplement (Thermo Fisher Scientific). Medium was changed every 2–3 days.

Organoids were passaged every 7 days. Matrigel domes were mechanically disrupted, collected in cold Advanced DMEM/F12, and centrifuged at 300 × *g* for 5 min. The pellet was resuspended in Matrigel and replated at a 1:2–1:4 split ratio.

Organoid formation was assessed after 7 days of culture at each passage, and structures > 100 μm in diameter were counted as organoids. Formation efficiency at subsequent passages was expressed relative to the number of organoids at P1, which was defined as 100%. Each animal‐derived organoid culture was considered one biological replicate; multiple Matrigel domes or images from the same culture were treated as technical replicates and were not considered independent samples.

### 2.10 Quantification of Organoid Size

Organoids were imaged using a BZ‐X800. Z‐stack images were acquired at ×2 magnification with 15 μm intervals to cover the entire Matrigel dome. Full‐focus images were generated using the built‐in software (Keyence). Organoid diameters were measured using ImageJ. All organoids within each Matrigel were included in the analysis.

### 2.11 Immunofluorescence Analysis of Organoid Sections

Organoids were harvested using Cultrex Organoid Harvesting Solution (R&D Systems, Minneapolis, MN, USA), fixed in 4% paraformaldehyde, embedded in O.C.T. compound, and cryosectioned at 20 μm. Sections were blocked with 1.5% normal goat serum and incubated with mouse anti‐p16 (1:50; Santa Cruz Biotechnology), rabbit anti‐p21 (1:200; Proteintech), mouse anti‐γH2AX (1:50; Santa Cruz Biotechnology), mouse anti‐Lamin B1 (1:100; Santa Cruz Biotechnology), or rabbit anti‐Ki67 (1:800; Abcam), followed by fluorescent secondary antibodies (1:1000; Thermo Fisher Scientific). Images were acquired using a BZ‐X800 microscope at ×10 magnification. Five organoids were randomly selected per animal and analyzed in a blinded manner. p16‐, p21‐, γH2AX‐, and Ki67‐positive cells were expressed as percentages of total DAPI‐positive nuclei, and Lamin B1 fluorescence intensity was normalized to the number of DAPI‐positive nuclei.

### 2.12 RNA Sequencing and Gene Ontology and Gene Set Enrichment Analyses

RNA sequencing (RNA seq), gene ontology (GO) and gene set enrichment analysis (GSEA) were performed as previously described (Ando et al. 2022). Total RNA from organoids was subjected to RNA seq (NovaSeq 6000 System; Illumina, San Diego, CA, USA), at depth of ~20 million reads per sample. Reads were aligned to the rat reference genome ( *Rattus norvegicus* ; Ensembl) using STAR aligner. Gene expression levels were quantified as read counts using RSEM. Differential gene expression analysis between the C‐ and HF‐derived organoids was performed using DESeq2 based on count data. Differentially expressed genes (DEGs) were defined as those with an adjusted *p*‐value < 0.05, calculated using the Benjamini–Hochberg method.

GO enrichment analysis of the DEGs was performed using Metascape. In addition, genes with a base mean expression value ≥ 15 were subjected to GSEA using the GSEA tool (version 4.1.0). The GO biological process (GOBP) gene sets were downloaded from the Molecular Signatures Database. For statistical significance testing, 1000 permutations were performed, and gene sets with a nominal *p*‐value (NOM *p*‐value) < 0.05 were considered statistically significant. The RNA‐seq data generated in this study have been deposited in the NCBI Gene Expression Omnibus (GEO) under accession number GSE348195. The complete list of DEGs, including gene symbols, gene IDs, baseMean values, log_2_ fold changes, and adjusted *p*‐values, is provided in Table S2.

### 2.13 Total RNA Isolation and Quantitative Reverse Transcription–Polymerase Chain Reaction

Total RNA was extracted from liver and organoid samples using the RNAiso Plus (Takara Bio, Otsu, Japan). Total RNA was reverse‐transcribed into cDNA using the ReverTra Ace qPCR RT Kit (TOYOBO, Osaka, Japan). The gene expression levels were normalized to β‐actin, and relative gene expression was calculated using the 2^−ΔΔ*Ct*^ method. Fold changes between the control and the HF‐derived organoids were determined. The primer sequences are listed in Table S1.

### 2.14 Flow Cytometric Analysis of Lipid Content in Liver Organoids

Organoids were treated with 400 μM oleic acid (OA) or vehicle control (BSA alone) for 48 h. Organoids were harvested and washed twice with Hanks' balanced salt solution (HBSS). The organoids were dissociated into single cells using TrypLE Express (Thermo Fisher Scientific) at 37°C for 30 min, with gentle trituration every 5 min. Cells were fixed with 4% paraformaldehyde for 15 min at room temperature and stained with BODIPY 493/503 (Thermo Fisher Scientific) at a final concentration of 0.5 μg/mL for 30 min at room temperature in the dark. Samples were analyzed using a flow cytometer (Gallios; Beckman Coulter, Brea, CA, USA). BODIPY 493/503 was excited with a 488 nm laser and fluorescent emission signals were collected using an FL1 (525/40 nm BP) detector. At least 5000 events were recorded per sample. Single cells were gated by FSC/SSC and singlet gating. BODIPY‐positive cells were defined using unstained control samples, and both the percentage of BODIPY‐positive cells and median fluorescence intensity (MFI) were quantified using Kaluza Analysis 2.2 software (Beckman Coulter). The percentage of positive cells was calculated as the number of BODIPY‐positive cells divided by the total number of cells counted. The rate of change was expressed as a fold increase relative to the percentage of positive cells in OA‐untreated cells in the C‐ and HF‐derived organoids.

### 2.15 Measurement of Reactive Oxygen Species in Organoids

Reactive oxygen species (ROS) levels were measured in organoids cultured for 7 days or additionally treated for 24 h with 800 μM OA and 400 μM palmitic acid (PA; Wako Pure Chemicals). Organoids were harvested and homogenized in RIPA buffer. The homogenized samples were subjected to ROS measurement using the OxiSelect In Vitro ROS Assay Kit (Cell Biolabs, San Diego, CA, USA). For the OA/PA–treated samples, untreated organoids cultured for 8 days in BSA‐containing medium were used as the reference control for normalization.

### 2.16 Measurement of Oxygen Consumption Rate in Organoids

Oxygen consumption rate (OCR) was measured as previously described (Yamada et al. 2019). Organoids were harvested and transferred to a 96‐well plate. Oxygen consumption was measured using the MitoXpress Oxygen Consumption Assay (Agilent Technologies, Santa Clara, CA, USA). Fluorescence intensity was recorded at 60‐s intervals for a total of 120 cycles using an Arvo X5, with excitation at 340 nm and emission at 642 nm. OCR values were normalized to total DNA content.

### 2.17 Measurement of Mitochondrial DNA (mtDNA) Content in Organoids

Total DNA was extracted from organoids using the NucleoSpin Tissue kit (Takara Bio) according to the manufacturer's instructions. The mtDNA content was quantified by real‐time PCR as previously described (Mizuno et al. 2023). The primer sequences used in this study are listed in Table S1. The relative mtDNA content was calculated using the 2^−ΔΔ*Ct*^ method, where Δ*Ct* was defined as *Ct*_mt‐Cox1_ − *Ct*_Rpl4_.

### 2.18 JC‐1 Staining of Liver Organoids

Mitochondrial membrane potential was assessed using the JC‐1 MitoMP Detection Kit (Dojindo, Kumamoto, Japan). Organoids were harvested and washed with pre‐warmed Advanced DMEM/F12 at 37°C, and incubated with JC‐1 working solution in organoid culture medium for 60 min at 37°C in 5% CO_2_. After washing, organoids were transferred to imaging buffer. Fluorescent images were acquired using a BZ‐X800.

### 2.19 Pharmacological Treatment of Liver Organoids

Organoids were initially cultured in standard organoid medium for 6 days (P1). After passaging, HF‐derived organoids were treated with additional N‐acetylcysteine (NAC; 1.25 mM; final concentration, 2.5 mM), BAY 11‐7082 (BAY; 5 μM), or mitoquinone (MitoQ; 100 nM). The same treatment conditions were maintained through P2 and P3. Untreated C‐ and HF‐derived organoids were cultured in parallel as controls. At P3, ROS levels, inflammatory cytokine secretion, OCR, and organoid formation efficiency were assessed as described above.

### 2.20 Statistical Analyses

Organoid formation efficiency is presented as mean ± standard error (SE), all other data as mean ± standard deviation (SD). Body weight and caloric intake were analyzed using Bonferroni's multiple comparison test. Survival curves for organoid formation were analyzed by Kaplan–Meier method with log‐rank test. Two group comparisons were performed using Student's *t*‐test. Comparisons among three or more groups were performed using one‐way analysis of variance (ANOVA) followed by Dunnett's multiple‐comparison test. For RNA seq data, DEGs were defined as adjusted *p*‐value < 0.05 using the Benjamini–Hochberg method. GSEA was performed with 1000 permutations. Gene sets with a NOM *p*‐value < 0.05 were considered statistically significant. Statistical analyses were performed using JMP software (version 14; SAS Institute, Cary, NC, USA). A *p*‐value < 0.05 was considered statistically significant.

## 3 Results

### 3.1 HFD Induces Hepatic Aging‐Like Dysfunction Under Chronic Metabolic Stress

The experimental design is shown in Figure 1A. Body weight was higher in the HF group than in the C group beginning at Week 4 after initiation of the HFD. Caloric intake was increased as early as 1 week after diet initiation (Figure 1B). At the end of the feeding period, liver weight did not differ between groups, whereas white adipose tissue (WAT) weight was increased in the HF group (Figure 1C). Serum analyses revealed significantly elevated T‐Cho and non‐HDL‐C levels in the HF group, whereas TG and HDL‐C levels were unchanged (Figure 1D).

**FIGURE 1:**
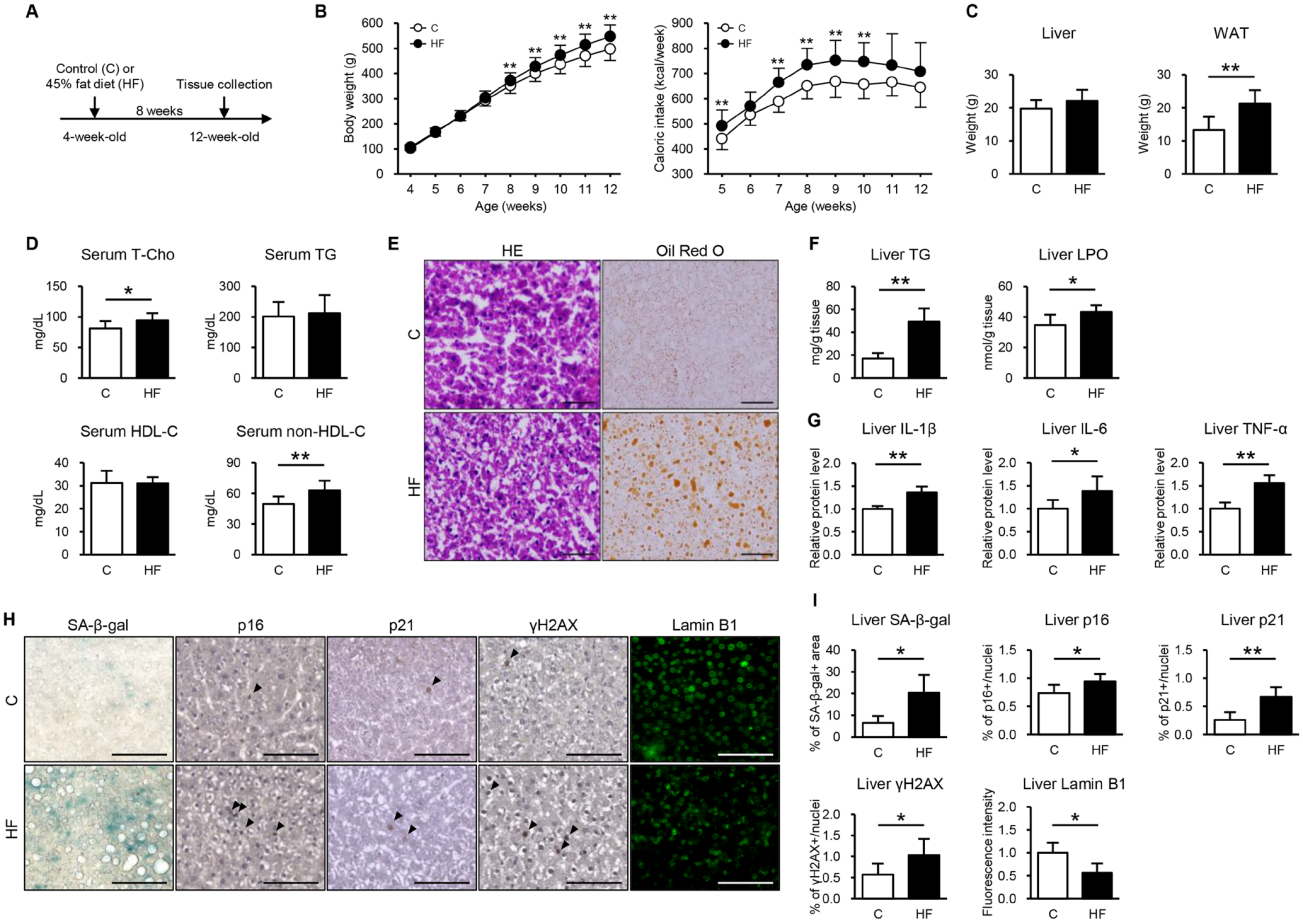
HFD induces aging‐like hepatic dysfunction under chronic metabolic stress. (A) Experimental design. (B) Body weight and caloric intake (*n* = 41–44/group). (C) Liver and white adipose tissue (WAT) weights (*n* = 9–11/group). (D) Serum total cholesterol (T‐Cho), HDL cholesterol (HDL‐C), non‐HDL cholesterol (non‐HDL‐C), and triglycerides (TG) (*n* = 11/group). (E) Representative hematoxylin–eosin (HE) and Oil Red O staining of liver sections. Scale bar, 50 μm. (F) Hepatic TG content and lipid peroxidation (LPO) levels (*n* = 6/group). (G) Hepatic inflammatory cytokine levels (IL‐1β, IL‐6, and TNF‐α) (*n* = 5/group). (H) Representative images of SA‐β‐gal staining and immunostaining for p16, p21, γH2AX, and Lamin B1 in liver sections. Arrows indicate positive cells. Scale bars, 100 μm. (I) Quantification of the SA‐β‐gal‐positive area, the numbers of p16‐, p21‐, and γH2AX‐positive cells, and Lamin B1 fluorescence intensity (*n* = 4–5/group). Data are presented as mean ± SD. Body weight and caloric intake were analyzed using Bonferroni's multiple comparison test. All other data were analyzed using Student's *t*‐test. *p < 0.05; **p < 0.01.

To determine whether chronic HFD feeding induced aging‐like hepatic dysfunction, we evaluated lipid accumulation, oxidative stress, inflammatory responses, and senescence‐associated markers in the liver. Oil Red O staining showed marked lipid accumulation in the HF group (Figure 1E), consistent with significantly increased hepatic triglyceride levels (Figure 1F). Hepatic lipid peroxide (LPO) was also significantly elevated, indicating enhanced oxidative stress (Figure 1F). Hepatic IL‐1β, IL‐6, and TNF‐α levels were significantly increased in the HF group (Figure 1G). The SA‐β‐gal‐positive area and the numbers of p16‐, p21‐, and γH2AX‐positive cells were significantly increased, whereas Lamin B1 fluorescence intensity was significantly reduced in the HF group (Figure 1H,I). Together, these findings indicate that chronic HFD‐induced metabolic stress promotes an aging‐like hepatic phenotype characterized by steatosis, oxidative stress, inflammatory activation, and accumulation of senescence‐associated cells.

### 3.2 HFD Impairs Regenerative Responses and Enhances Senescence‐Associated Changes After Liver Injury

We next examined how chronic metabolic stress influenced regenerative responses after liver injury using a CCl_4_‐induced injury model (Figure 2A,B). In this context, facultative regenerative programs involving liver stem/progenitor cells have been reported to contribute, at least in part, to epithelial repair (Huch et al. 2013). To assess regenerative activity, we quantified Ki67‐positive cells as a marker of proliferative repair. The proportion of Ki67‐positive cells was significantly lower in the HF group than in the C group (Figure 2C), indicating impaired regenerative responses under chronic HFD‐induced metabolic stress.

**FIGURE 2:**
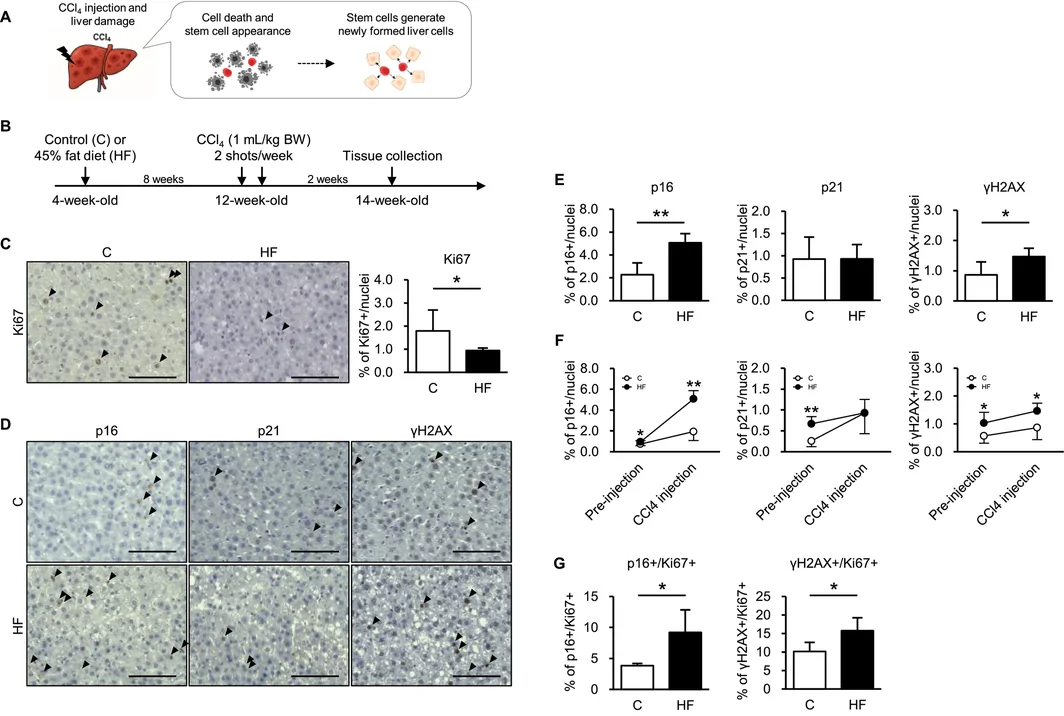
HFD impairs regenerative responses and enhances senescence‐associated changes after CCl_4_‐induced liver injury. (A) CCl_4_‐induced liver injury and regeneration model. In this injury context, facultative regenerative programs involving liver stem/progenitor cells have been reported to contribute, at least in part, to epithelial repair. (B) Eight weeks of dietary feeding followed by two CCl_4_ injections within 1 week (1 mL/kg body weight diluted 1:1 in olive oil). Regenerating livers were assessed 2 weeks after the final injection. (C) Representative staining images and quantification of the percentage of Ki67‐positive cells in regenerating livers (% nuclei; *n* = 3–6 per group). (D) Representative staining images of p16, p21, and γH2AX in regenerating liver tissue. Arrows indicate positive cells. Scale bar: 100 μm. (E) Quantification of the percentage of senescence marker‐positive cells in regenerating livers (% nuclei; *n* = 3–6/group). (F) Temporal changes in the percentages of p16‐, p21‐, and γH2AX‐positive cells before and after CCl_4_ injury (*n* = 3–6/group). (G) p16‐ and γH2AX‐positive cells among Ki67‐positive cells in regenerating livers (*n* = 3–6/group). Data are presented as mean ± SD. *p < 0.05; **p < 0.01, Student's *t*‐test.

Representative immunostaining images of senescence‐associated markers are shown in Figure 2D. The proportions of p16‐ and γH2AX‐positive cells were significantly increased in the HF group, whereas p21 positivity did not differ between groups (Figure 2E). The proportions of p16‐, p21‐, and γH2AX‐positive cells increased after CCl_4_ administration in both groups (Figure 2F). To determine whether senescence‐associated changes accompanied regenerative proliferation, serial liver sections were stained for Ki67 together with p16 or γH2AX. Following CCl_4_ administration, the proportions of p16‐positive and γH2AX‐positive cells among Ki67‐positive cells were significantly increased in the HF group (Figure 2G). These findings indicate that chronic metabolic stress impairs regenerative repair and promotes the accumulation of senescence‐associated cells during liver regeneration.

### 3.3 Liver Organoids From HFD‐Fed Rats Retain Impaired Regenerative Capacity and a Persistent Senescence‐Like Phenotype

To determine whether chronic metabolic stress induces persistent abnormalities in regenerative liver cell populations, we established liver organoids from control and HFD‐fed rats (Figure 3A). Representative images of primary organoids are shown in Figure 3B. The total number of organoids measuring > 100 μm in diameter tended to be lower in the HF‐derived organoids (Figure 3C). Analysis of organoid size distribution revealed an enrichment of smaller organoids (100–400 μm) in the HF‐derived organoids, whereas the proportion of large organoids (> 400 μm) was significantly higher in C‐derived organoids (Figure 3C). Consistently, both the maximum and the mean diameter of the five largest organoids per Matrigel dome were significantly smaller in the HF‐derived organoids (Figure 3C).

**FIGURE 3:**
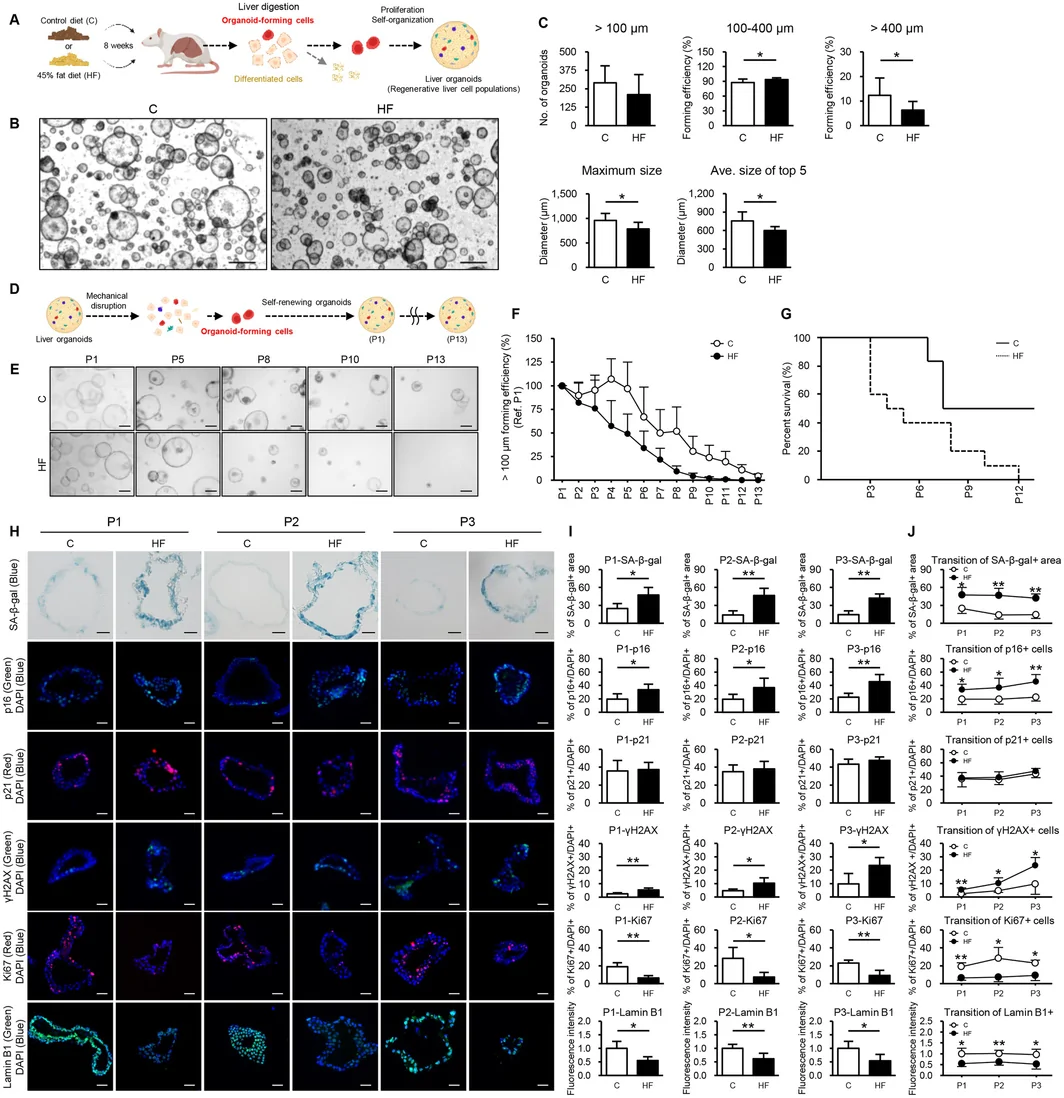
Liver organoids from HFD‐fed rats exhibit reduced formation capacity and a persistent senescence‐associated phenotype. (A) Liver tissues from control and HFD‐fed rats were dissociated, and regenerative cell–enriched fractions were cultured in Matrigel domes to generate organoids. (B) Representative images of primary organoids. Scale bar, 500 μm. (C) Organoid size distribution and diameter (*n* = 6–10/group). (D) Organoids were mechanically disrupted and replated at each passage to assess self‐renewal capacity. (E) Representative images of organoids at Passages 1 (P1), 5, 8, 10, and 13. Scale bar, 200 μm. (F) Organoid formation efficiency during serial passaging. The formation rate is expressed as a percentage of the number of P1 organoids and is normalized to 100% (*n* = 6–10/group). (G) Kaplan–Meier survival analysis of organoid formation. Loss of organoid formation was defined as the absence of organoids measuring > 100 μm in diameter (*n* = 6–10/group). (H) Representative images of SA‐β‐gal staining and immunofluorescence staining for p16, p21, γH2AX, Ki67, and Lamin B1 in organoid sections at P1–3. DAPI (blue) labels nuclei. Green fluorescence indicates p16, γH2AX, and Lamin B1, whereas red fluorescence indicates p21 and Ki67. Scale bar, 50 μm. (I) Quantification of the SA‐β‐gal‐positive area, the numbers of p16‐, p21‐, γH2AX‐, and Ki67‐positive cells, and Lamin B1 fluorescence intensity at each passage (*n* = 4–6/group). (J) Passage‐dependent changes in each senescence‐associated marker (*n* = 4–6/group). Each animal‐derived organoid culture was considered one biological replicate. Formation efficiency is presented as mean ± SE; all other data are presented as mean ± SD. Survival curves were analyzed using the Kaplan–Meier method, and statistical significance was determined using the log‐rank test. All other data were analyzed using Student's *t*‐test. *p < 0.05; **p < 0.01.

To assess regenerative capacity ex vivo, organoids were serially passaged up to Passage 13 (P13) (Figure 3D,E). When organoid formation at Passage 1 (P1) was normalized to 100%, HF‐derived organoids exhibited reduced formation efficiency beginning at P3 (Figure 3F). Kaplan–Meier analysis, in which the absence of organoids measuring > 100 μm was defined as loss of organoid formation, revealed markedly reduced serial propagation capacity of HF‐derived organoids (Figure 3G). We next examined whether senescence‐associated alterations were retained during serial propagation. Representative staining images are shown in Figure 3H. HF‐derived organoids exhibited increased SA‐β‐gal‐positive areas and higher proportions of p16‐ and γH2AX‐positive cells, together with reduced Ki67 positivity and Lamin B1 fluorescence intensity (Figure 3I). These senescence‐associated differences remained detectable through P2 and P3 (Figure 3I,J). Together, these findings demonstrate that impaired regenerative capacity and a broad senescence‐like phenotype persist in HF‐derived organoids after removal from the in vivo metabolic environment and during serial propagation.

### 3.4 Transcriptomic Profiling Identifies Inflammaging‐ and Mitochondrial Dysfunction‐Related Signatures in HF‐Derived Organoids

To characterize senescence‐associated molecular changes in HF‐derived organoids, RNA seq was performed. Scatter plot and volcano plot analyses identified 29 upregulated and 71 downregulated genes in HF‐derived organoids (Figure S1A,B; Table S2). Among these genes, *Gpnmb*, a senescence‐associated glycoprotein (Suda et al. 2021), was one of the most significantly upregulated (Figure S1B). GSEA revealed significant enrichment of pathways related to lipid metabolism, inflammation, oxidative stress, and mitochondrial function in HF‐derived organoids (Figure S1C). Consistently, hepatic *Gpnmb* expression was significantly elevated in vivo in the HF group (Figure S2A), further supporting the organoid‐based findings. These transcriptomic data indicate that chronic HFD‐induced metabolic stress imprints a senescence‐associated transcriptional program in liver organoids, including signatures related to inflammaging and mitochondrial dysfunction.

### 3.5 HF‐Derived Liver Organoids Exhibit Enhanced Oxidative Stress and Inflammatory/SASP‐Associated Responses

GSEA revealed significant enrichment of the *SUPEROXIDE ANION GENERATION* pathway in HF‐derived organoids (Figure 4A). qPCR analysis confirmed upregulation of *Itgb2* and *Cybb*, indicating enhanced oxidative stress signaling (Figure 4A). Genes in the *POSITIVE REGULATION OF INTERLEUKIN‐6 PRODUCTION* pathway, including *Clec7a*, *Tlr3*, and *Il1rl2*, were also upregulated in HF‐derived organoids (Figure 4B). In vivo, *Itgb2* and *Clec7a* were increased in the HF group, whereas *Cybb* and *Tlr3* did not differ between the groups (Figure S2B).

**FIGURE 4:**
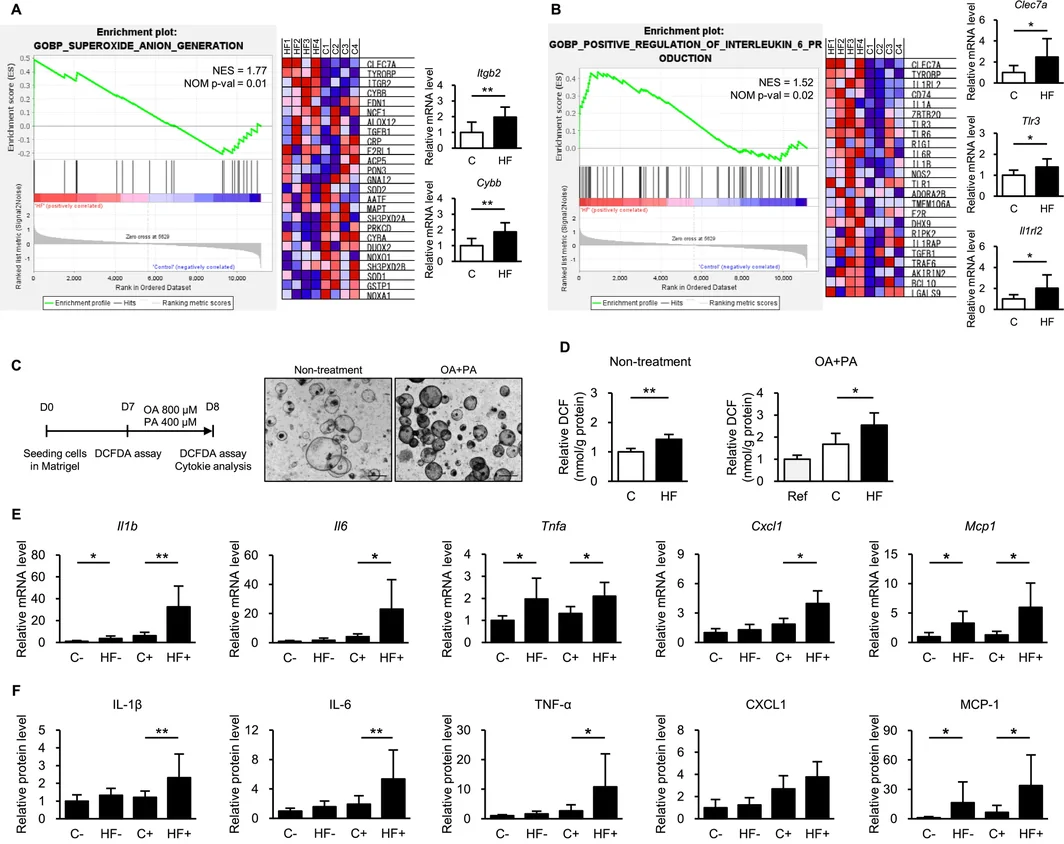
HF‐derived liver organoids display enhanced oxidative stress and inflammatory responses. (A) Gene set enrichment analysis (GSEA) enrichment plot and quantitative PCR (qPCR) validation of the *SUPEROXIDE ANION GENERATION* pathway (GSEA enrichment plot: *n* = 4/group; qPCR validation: *n* = 8–11/group). (B) GSEA enrichment plot and qPCR validation of the *POSITIVE REGULATION OF INTERLEUKIN‐6 PRODUCTION* pathway (GSEA enrichment plot: *n* = 4/group; qPCR validation: *n* = 8–11/group). (C) Organoids were exposed to a combination of fatty acids (800 μM oleic acid [OA] and 400 μM palmitic acid [PA]) for 24 h, followed by analysis of intracellular reactive oxygen species (ROS) and inflammatory cytokines. Representative images of non‐treated and OA + PA‐treated organoids are shown. Scale bar, 500 μm. (D) Intracellular ROS levels at baseline (left) and after OA + PA treatment (right) (*n* = 6/group). Non‐treated organoids cultured in parallel with the fatty acid exposure group were used as a reference (Ref; *n* = 3). (E) Gene expression levels of *Il1b*, *Il6*, *Tnfa*, *Cxcl1*, and *Mcp1* (*n* = 5–6/group). (F) Quantification of secreted IL‐1β, IL‐6, TNF‐α, CXCL1, and MCP‐1 in organoid culture supernatants (*n* = 7–8/group). Data are presented as mean ± SD. *p < 0.05; **p < 0.01, Student's *t*‐test.

Intracellular ROS levels were significantly elevated in HF‐derived organoids at baseline and remained higher following combined OA and PA exposure (Figure 4C,D). Following fatty acid exposure, inflammatory cytokine and chemokine mRNA levels (*Il1b*, *Il6*, *Tnfa*, *Cxcl1*, and *Mcp1*) were significantly increased in HF‐derived organoids (Figure 4E). Consistently, secretion of IL‐1β, IL‐6, TNF‐α, and MCP‐1 was significantly increased, whereas CXCL1 secretion did not differ significantly between groups (Figure 4F). Together, these findings demonstrate that HF‐derived organoids exhibit enhanced oxidative stress and a broad inflammatory/SASP‐associated response.

### 3.6 HF‐Derived Liver Organoids Exhibit Enhanced Lipid Accumulation Capacity and Mitochondrial Dysfunction

To validate the transcriptional changes related to lipid metabolism and mitochondrial function, we examined genes included in the *FATTY ACID TRANSPORT* and *MITOCHONDRIAL GENE EXPRESSION* gene sets (Figure 5A,B). qPCR analysis confirmed the upregulation of *Thbs1*, *Acsl3*, and *Plin2* and the downregulation of *Mto1*, *Mrps24*, and *Tfam* in HF‐derived organoids, consistent with the RNA‐seq results (Figure 5A,B). Similar expression changes were observed in vivo (Figure S2C). We additionally examined genes more directly involved in fatty acid uptake, triglyceride synthesis, and de novo lipogenesis. Expression of the fatty acid uptake‐related genes *Cd36* and *Fatp1* was significantly increased in HF‐derived organoids (Figure 5C and Figure S2D). In contrast, expression of the de novo lipogenesis‐related genes *Dgat1*, *Fasn*, *Acaca*, *Elovl6*, and *Scd1* did not differ significantly between C‐ and HF‐derived organoids (Figure 5C and Figure S2D). These findings indicate that the altered lipid‐handling phenotype of HF‐derived organoids is accompanied by increased expression of fatty acid uptake‐related genes without significant differences in the expression of the de novo lipogenesis‐related genes examined.

**FIGURE 5:**
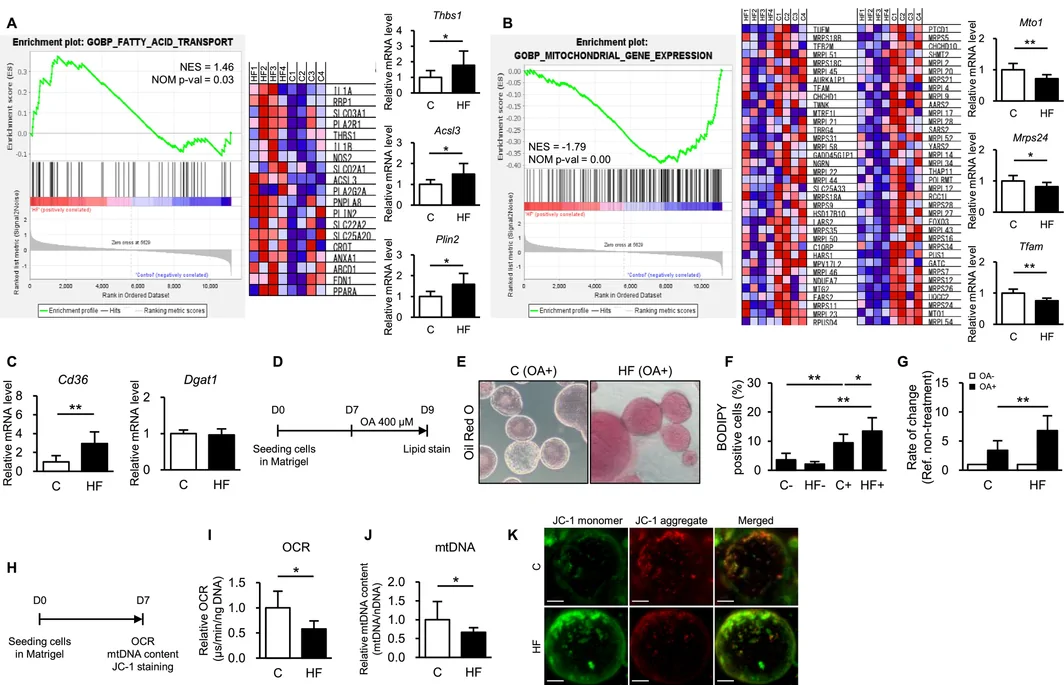
HF‐derived liver organoids exhibit enhanced lipid accumulation capacity and mitochondrial dysfunction. (A) Gene expression profile of the *FATTY ACID TRANSPORT* gene set and qPCR validation of *Thbs1*, *Acsl3*, and *Plin2* (GSEA enrichment plot: *n* = 4/group; qPCR validation: *n* = 8–11/group). (B) Gene expression profile of the *MITOCHONDRIAL GENE EXPRESSION* gene set and qPCR validation of *Mto1*, *Mrps24*, and *Tfam* (GSEA enrichment plot: *n* = 4/group; qPCR validation: *n* = 8–11/group). (C) Gene expression levels of *Cd36* and *Dgat1* (*n* = 8/group). (D) Organoids were exposed to 400 μM oleic acid (OA) or vehicle control (BSA alone) for 48 h, followed by analysis of lipid accumulation. (E) Representative images of Oil Red O staining. (F) Flow cytometric analysis of BODIPY staining (*n* = 6/group). (G) Fold change in BODIPY‐positive cells relative to the non‐treated baseline in each group (*n* = 6/group). (H) Experimental design for mitochondrial function assessment. (I) Oxygen consumption rate (OCR) measured in liver organoids (*n* = 6/group). (J) mtDNA content quantified by qPCR using mitochondrial‐specific primers (*Cox1*) relative to nuclear DNA (*Rpl4*) (*n* = 8/group). (K) Representative JC‐1 staining images. JC‐1 monomers are shown in green and aggregates in red. Scale bar, 100 μm. Data are presented as mean ± SD. *p < 0.05; **p < 0.01, Student's *t*‐test.

We next evaluated lipid accumulation following OA exposure (Figure 5D). Oil Red O staining showed increased lipid accumulation in HF‐derived organoids compared with C‐derived organoids (Figure 5E). BODIPY analysis showed that OA increased the proportion of lipid‐positive cells in both groups, with a significantly greater increase in HF‐derived organoids (Figure 5F). Consistently, the fold increase in lipid accumulation relative to baseline was greater in HF‐derived organoids (Figure 5G). Together, these findings indicate that HF‐derived organoids retain an enhanced capacity to accumulate exogenous lipid, accompanied by increased *Cd36* and *Fatp1* expression.

We subsequently assessed mitochondrial function (Figure 5H). HF‐derived organoids exhibited reduced OCR and mtDNA content compared with C‐derived organoids (Figure 5I,J). JC‐1 staining further showed increased monomer fluorescence and decreased aggregate fluorescence in HF‐derived organoids, consistent with mitochondrial membrane depolarization (Figure 5K). Together, these findings demonstrate persistent mitochondrial dysfunction in HF‐derived organoids.

### 3.7 Pharmacological Improvement of Mitochondrial Dysfunction Partially Restores Regenerative Capacity in HF‐Derived Liver Organoids

To examine whether mitochondrial dysfunction functionally contributes to the impaired regenerative capacity of HF‐derived organoids, we treated HF‐derived organoids with the mitochondria‐targeted antioxidant MitoQ during serial propagation (Figure 6A). Untreated HF‐derived organoids exhibited significantly elevated ROS levels and increased secretion of TNF‐α and MCP‐1 compared with C‐derived organoids. MitoQ treatment significantly attenuated these abnormalities (Figure 6B,C).

**FIGURE 6:**
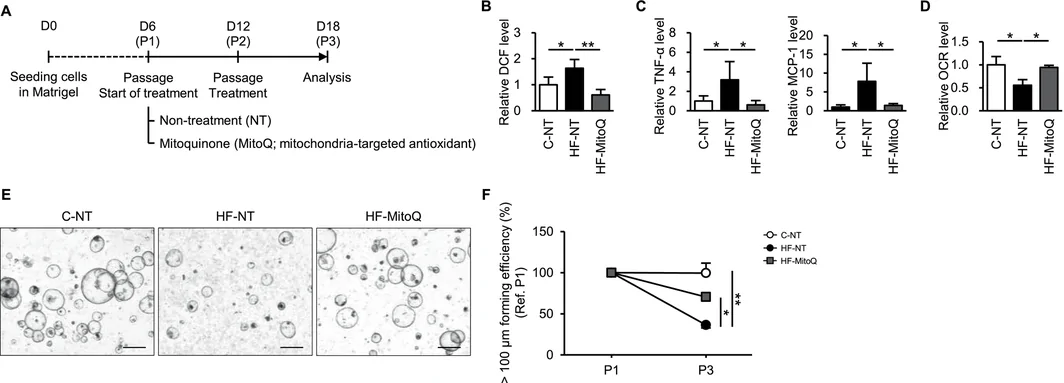
MitoQ improves mitochondrial function and partially restores organoid formation efficiency in HF‐derived liver organoids. (A) Experimental design. Organoids were initially cultured in standard organoid medium for 6 days at Passage 1 (P1). After passaging, HF‐derived organoids were treated with mitoquinone (MitoQ; 100 nM) throughout P2 and P3. Untreated control (C)‐ and HF‐derived organoids were cultured in parallel. (B) Relative intracellular DCF levels at P3 (*n* = 4–5/group). (C) Relative levels of secreted TNF‐α and MCP‐1 at P3 (*n* = 4–6/group). (D) Relative oxygen consumption rate (OCR) at P3 (*n* = 3/group). (E) Representative images of organoids at P3. Scale bar, 500 μm. (F) Organoid formation efficiency at P1 and P3 (*n* = 6/group). Formation efficiency is expressed relative to the number of organoids at P1, which was defined as 100%. Each animal‐derived organoid culture was considered one biological replicate. Organoid formation efficiency (F) is presented as mean ± SE; all other quantitative data are presented as mean ± SD. Statistical significance was assessed using one‐way ANOVA followed by Dunnett's multiple‐comparison test, with the HF‐NT group as the control. *p < 0.05; **p < 0.01.

OCR was significantly reduced in untreated HF‐derived organoids, whereas MitoQ treatment increased OCR to a level comparable to that observed in C‐derived organoids (Figure 6D). We next examined whether these improvements were accompanied by recovery of regenerative capacity. Organoid formation efficiency at P3 was significantly reduced in untreated HF‐derived organoids compared with C‐derived organoids. MitoQ treatment significantly increased formation efficiency compared with untreated HF‐derived organoids, although recovery remained incomplete relative to C‐derived organoids (Figure 6F).

In parallel, we also examined NAC and BAY 11‐7082 as interventions targeting oxidative stress and inflammatory signaling, respectively. Although these treatments affected selected stress‐related readouts, neither significantly improved organoid formation efficiency under the conditions examined (Figure S3). Together, these findings support a functional contribution of mitochondrial dysfunction to the regenerative impairment of HF‐derived organoids, although MitoQ did not fully correct the regenerative deficit under the conditions examined.

## 4 Discussion

The present study identifies durable senescence‐like reprogramming of the liver regenerative compartment as a consequence of chronic HFD‐induced metabolic stress. In vivo, HFD feeding induced steatosis, oxidative stress, inflammatory activation, and multiple senescence‐associated changes, accompanied by reduced proliferative responses following CCl_4_‐induced liver injury. Importantly, HF‐derived organoids retained impaired formation and serial propagation capacity, with senescence‐associated abnormalities remaining detectable across successive passages outside the original metabolic environment. These cultures also exhibited altered lipid handling, enhanced oxidative and inflammatory responses, and mitochondrial dysfunction. MitoQ treatment improved OCR and partially restored organoid formation efficiency, supporting a functional contribution of mitochondrial dysfunction to the regenerative defect. Together, these findings extend the effects of chronic metabolic stress beyond abnormalities in the exposed liver to persistent dysfunction retained within organoid‐forming regenerative cell populations.

Previous studies have established hepatocyte senescence as an important feature of metabolic liver injury (Aravinthan et al. 2013; Ogrodnik et al. 2017). The principal advance of the present study is therefore not the association between HFD and hepatic senescence itself, but the finding that impaired organoid formation and senescence‐associated features persist within organoid‐forming regenerative cell populations after removal from the original metabolic environment and during serial propagation. Liver progenitor populations can contribute to epithelial repair under defined injury conditions (Lu et al. 2015; Pu et al. 2023), raising the possibility that lasting changes in their functional state influence long‐term tissue integrity. We use durable reprogramming here to describe a persistent alteration in the functional state of the organoid‐forming regenerative population, consistent with a cellular memory of prior metabolic stress. This perspective complements the hepatocyte‐centered framework by recognizing that metabolic stress may leave lasting consequences not only in existing tissue cells but also in populations capable of regenerative expansion.

The senescence‐like interpretation is supported by complementary cellular and functional findings rather than by any single marker. HF‐derived organoids showed increased SA‐β‐gal staining and p16 and γH2AX positivity, together with reduced Ki67 positivity and Lamin B1 staining. These findings encompass changes in lysosomal activity, DNA damage‐associated signaling, proliferation, and nuclear lamina integrity. Enhanced inflammatory and SASP‐associated secretion following fatty acid challenge further indicated heightened stress responsiveness. Although individual markers did not change uniformly across conditions or passages, their combined pattern, together with impaired organoid formation and propagation, supports a broad senescence‐associated phenotype. Cellular senescence is itself a heterogeneous state characterized by persistent proliferative arrest together with variable stress‐response and secretory features (Campisi and D'Adda Di Fagagna 2007). We therefore use “senescence‐like” because irreversible cell‐cycle arrest was not established at the single‐cell level and the cultures should not be regarded as a uniform population of senescent cells. A direct comparison with other senescence‐inducing stimuli was also not performed, so the data do not establish an HFD‐specific senescence program. In contrast, “aging‐like” is used more broadly to describe the combined metabolic, oxidative, inflammatory, senescence‐associated, and regenerative abnormalities observed at the tissue level (López‐Otín et al. 2023; Ogrodnik et al. 2017), without implying equivalence to naturally aged liver tissue.

Our data identify mitochondrial dysfunction as a prominent feature of the persistent senescence‐like state in HF‐derived organoids. Reduced OCR and mtDNA content, downregulation of mitochondrial regulatory genes, and JC‐1 staining consistent with membrane depolarization collectively indicate disrupted mitochondrial homeostasis. Mitochondrial dysfunction and cellular senescence can reinforce one another through changes in cellular metabolism and stress signaling (Miwa et al. 2022). Disturbed mitochondrial homeostasis can promote senescence‐associated phenotypes, while mitochondrial stress in senescent cells can sustain inflammatory signaling (Victorelli et al. 2023; Wiley et al. 2016). In this context, the coexistence of mitochondrial dysfunction, oxidative stress, and inflammatory activation in HF‐derived organoids is consistent with a self‐reinforcing interplay that could help maintain the persistent dysfunctional state. Importantly, MitoQ treatment reduced ROS levels and TNF‐α and MCP‐1 secretion, improved OCR, and partially restored organoid formation efficiency during serial propagation. The accompanying improvement in regenerative capacity provides pharmacological support for a functional contribution of mitochondrial dysfunction, extending the associations identified in untreated organoids. In contrast, additional NAC supplementation reduced the measured ROS signal without significantly improving organoid formation, whereas BAY 11‐7082 likewise did not significantly improve formation under the conditions examined. These findings indicate that improvement in the measured ROS readout did not necessarily translate into regenerative recovery, but do not exclude contributions from oxidative stress or inflammation. Because MitoQ affected respiratory, redox, and inflammatory readouts simultaneously, the intervention does not isolate a single causal pathway, and improved formation should not be equated with reversal of the entire senescence‐like state. Together, these results support mitochondria‐linked dysfunction as a pharmacologically modifiable component of the persistent regenerative defect.

The biological consequences of durable reprogramming within the regenerative compartment may extend beyond reduced regenerative output to altered functional properties of newly generated progeny. Studies in other tissues have linked altered stem/progenitor states to abnormal differentiation and inflammatory tissue remodeling (Ambrosi et al. 2021; Stearns‐Reider et al. 2017; Yan et al. 2018). In the liver, regenerative cells that retain proliferative capacity while carrying persistent abnormalities could similarly generate progeny predisposed to metabolic dysfunction, inflammatory activation, or senescence‐like states. This possibility is particularly relevant to recovery after metabolic improvement: reducing the original metabolic burden may not fully restore tissue maintenance if abnormal states remain within the cells responsible for tissue renewal. Persistent obesity‐associated transcriptional changes in human and mouse adipose tissue after weight loss (Hinte et al. 2024) and sustained hepatic DNA damage response signaling after dietary reversal in experimental steatohepatitis (Zou et al. 2024) illustrate that some cellular alterations can outlast improvement of the metabolic environment. Such persistence raises the possibility that residual cellular abnormalities may limit sustained recovery or increase susceptibility to renewed metabolic liver injury. Our organoid findings support persistence of an abnormal state during regenerative expansion, but do not establish that individual reprogrammed cells generate senescent differentiated descendants. Nevertheless, they motivate a model in which the regenerative compartment influences long‐term tissue integrity not only through the amount of tissue regenerated but also through the functional quality of the progeny it produces.

This study has several limitations. First, the precise cellular origin and composition of the organoid‐forming populations were not established, and persistence was demonstrated at the population rather than the individual‐cell level. Second, the experiments were restricted to male rats and a single HFD regimen. Third, the temporal sequence and the molecular mechanisms that establish and maintain the persistent phenotype remain incompletely defined. Finally, although MitoQ partially improved organoid formation during treatment, we did not determine whether this benefit persists after treatment withdrawal or translates into improved tissue repair in vivo.

In summary, this study identifies durable senescence‐like reprogramming of the liver regenerative compartment as a previously underappreciated consequence of chronic metabolic stress. Regenerative impairment and senescence‐associated abnormalities persisted during serial organoid propagation outside the original tissue environment, while partial recovery with MitoQ supports mitochondrial dysfunction as one functional component of the regenerative defect. This work therefore provides a conceptual framework linking metabolic liver aging to sustained dysfunction of the regenerative compartment and highlights this compartment as a potential target for future mechanistic and therapeutic investigation.

## Author Contributions

M.Y.: conceptualization, investigation, visualization, writing – original draft. H.Y.: conceptualization, supervision, writing – review and editing, funding acquisition. E.M.: conceptualization, writing – original draft. Y.A. and G.M.: investigation, visualization. T.W., T.S., K.H., H. Ichikawa, R.S., M.A., and H. Ishikawa: investigation. K.S.: formal analysis. K.O.: writing – review and editing. N.O.: writing – review and editing.

## Funding

This work was supported by Japan Society for the Promotion of Science (JSPS) (grant numbers JP24K02692 and JP24K20712).

## Ethics Statement

All experimental procedures were approved by the Animal Experiment Committee of Fujita Health University and were conducted in accordance with the institutional guidelines for the care and use of laboratory animals (approval number: APU19096).

## Conflicts of Interest

The authors declare no conflicts of interest.

## Supporting information


**Figure S1:** Transcriptomic profiling identifies inflammaging‐ and mitochondrial dysfunction‐related signatures in high‐fat diet fed rat‐derived liver organoids. (A) Scatter plot comparing gene expression between C and HF‐derived organoids (log_2_ scale). Each point represents a differentially expressed gene (DEG); red points represent upregulated genes, and blue points represent downregulated genes with an adjusted *p*‐value of less than 0.05 (*n* = 4/group). (B) Volcano plot visualization of DEGs showing log_2_ fold‐change (*x*‐axis) versus –log_10_ adjusted *p*‐value (*y*‐axis). Twenty‐nine genes were upregulated (red) and 71 genes were downregulated (blue) in HF‐derived organoids (*n* = 4/group). *Gpnmb* (senescence‐associated glycoprotein) is highlighted as one of the most significantly upregulated genes. (C) Gene set enrichment analysis (GSEA) showing significant enrichment of pathways related to lipid metabolism, inflammation, oxidative stress, and mitochondrial function in HF‐derived organoids (*n* = 4/group).
**Figure S2:** In vivo validation of transcriptomic alterations and additional analysis of lipid‐handling genes in HF‐derived liver organoids. (A) Quantitative PCR (qPCR) analysis of senescence‐related genes *Gpnmb* in liver tissue from control (C) and HFD‐fed (HF) rats (*n* = 5/group). (B) qPCR analysis of selected oxidative stress‐ and inflammation‐related genes in liver tissue from control (C) and HFD‐fed (HF) rats (*n* = 13/group). (C) qPCR analysis of selected lipid metabolism‐ and mitochondrial function‐related genes in liver tissue from C and HF rats (*n* = 13/group). (D) Expression of the fatty acid uptake‐related gene *Fatp1* and de novo lipogenesis‐related genes *Fasn*, *Acaca*, *Elovl6*, and *Scd1* in C‐ and HF‐derived liver organoids (*n* = 8/group). *Fatp1* expression was significantly increased in HF‐derived organoids, whereas *Fasn*, *Acaca*, *Elovl6*, and *Scd1* did not differ significantly between groups. Data are presented as mean ± SD. Statistical significance was assessed using Student's *t*‐test. *p < 0.05; **p < 0.01.
**Figure S3:** Effects of additional NAC supplementation and BAY 11‐7082 on stress‐related responses and organoid formation in HF‐derived liver organoids. (A) Experimental design. Organoids were initially cultured in standard organoid medium for 6 days at Passage 1 (P1). After passaging, HF‐derived organoids were treated with additional N‐acetylcysteine (NAC; 1.25 mM, resulting in a final concentration of 2.5 mM) or BAY 11‐7082 (BAY; 5 μM) throughout P2 and P3. Untreated control (C)‐ and HF‐derived organoids were cultured in parallel. (B) Relative intracellular DCF levels at P3 (*n* = 4–5/group). (C) Relative levels of secreted TNF‐α and MCP‐1 at P3 (*n* = 4–6/group). (D) Relative oxygen consumption rate (OCR) at P3 (*n* = 3/group). (E) Representative images of organoids at P3. Scale bar, 500 μm. (F) Organoid formation efficiency at P1 and P3 (*n* = 6/group). Formation efficiency is expressed relative to the number of organoids at P1, which was defined as 100%. Each animal‐derived organoid culture was considered one biological replicate. The same C‐NT and HF‐NT control data are shown in Figure 6 and Figure S3 to facilitate comparison. Organoid formation efficiency (F) is presented as mean ± SE; all other quantitative data are presented as mean ± SD. Statistical significance was assessed using one‐way ANOVA followed by Dunnett's multiple‐comparison test. *p < 0.05; **p < 0.01.

**Table S1:** Sequences of the primers.

**Table S2:** Differentially expressed genes in HF‐derived liver organoids compared with C‐derived liver organoids.

## Data Availability

The RNA‐seq data have been deposited in the NCBI Gene Expression Omnibus (GEO) under accession number GSE348195. The data that support the findings of this study are available from the corresponding author upon reasonable request.
