# Supplementary material for: High‐Fat Diet–Induced Metabolic Stress Durably Reprograms the Liver Regenerative Compartment Toward a Senescence‐Like State
Source: Aging Cell. 2026 Oct 16;25(10):e70769. doi: 10.1111/acel.70769 (PMC13673232; doi:10.1111/acel.70769)
Supplement: Supplementary file 1 [file ACEL-25-e70769-s002.docx]

**Figure S1.**  Transcriptomic profiling identifies inflammaging- and mitochondrial dysfunction-related signatures in high-fat diet fed rat-derived liver organoids. (A) Scatter plot comparing gene expression between C and HF-derived organoids (log₂ scale). Each point represents a differentially expressed gene (DEG); red points represent upregulated genes, and blue points represent downregulated genes with an adjusted p-value of less than 0.05 (n = 4/group). (B) Volcano plot visualization of DEGs showing log₂ fold-change (x-axis) versus −log₁₀ adjusted p-value (y-axis). Twenty-nine genes were upregulated (red) and 71 genes were downregulated (blue) in HF-derived organoids (n = 4/group). *Gpnmb* (senescence-associated glycoprotein) is highlighted as one of the most significantly upregulated genes. (C) Gene set enrichment analysis (GSEA) showing significant enrichment of pathways related to lipid metabolism, inflammation, oxidative stress, and mitochondrial function in HF-derived organoids (n = 4/group).

**Figure S2.** In vivo validation of transcriptomic alterations and additional analysis of lipid-handling genes in HF-derived liver organoids. (A) Quantitative PCR (qPCR) analysis of senescence-related genes *Gpnmb* in liver tissue from control (C) and HFD-fed (HF) rats (n = 5/group). (B) qPCR analysis of selected oxidative stress- and inflammation-related genes in liver tissue from control (C) and HFD-fed (HF) rats (n = 13/group). (C) qPCR analysis of selected lipid metabolism- and mitochondrial function-related genes in liver tissue from C and HF rats (n = 13/group). (D) Expression of the fatty acid uptake-related gene *Fatp1* and de novo lipogenesis-related genes *Fasn*, *Acaca*, *Elovl6*, and *Scd1* in C- and HF-derived liver organoids (n = 8/group). *Fatp1* expression was significantly increased in HF-derived organoids, whereas *Fasn*, *Acaca*, *Elovl6*, and *Scd1* did not differ significantly between groups. Data are presented as mean ± SD. Statistical significance was assessed using Student’s t-test. *p < 0.05; **p < 0.01.

**Figure S3.** Effects of additional NAC supplementation and BAY 11-7082 on stress-related responses and organoid formation in HF-derived liver organoids. (A) Experimental design. Organoids were initially cultured in standard organoid medium for 6 days at passage 1 (P1). After passaging, HF-derived organoids were treated with additional N-acetylcysteine (NAC; 1.25 mM, resulting in a final concentration of 2.5 mM) or BAY 11-7082 (BAY; 5 μM) throughout P2 and P3. Untreated control (C)- and HF-derived organoids were cultured in parallel. (B) Relative intracellular DCF levels at P3 (n = 4–5/group). (C) Relative levels of secreted TNF-α and MCP-1 at P3 (n = 4–6/group). (D) Relative oxygen consumption rate (OCR) at P3 (n = 3/group). (E) Representative images of organoids at P3. Scale bar, 500 µm. (F) Organoid formation efficiency at P1 and P3 (n = 6/group). Formation efficiency is expressed relative to the number of organoids at P1, which was defined as 100%. Each animal-derived organoid culture was considered one biological replicate. The same C-NT and HF-NT control data are shown in Figure 6 and Figure S3 to facilitate comparison. Organoid formation efficiency (F) is presented as mean ± SE; all other quantitative data are presented as mean ± SD. Statistical significance was assessed using one-way ANOVA followed by Dunnett’s multiple-comparison test. *p < 0.05; **p < 0.01.
